# Transcriptomic Immune Response of the Cotton Stainer *Dysdercus fasciatus* to Experimental Elimination of Vitamin-Supplementing Intestinal Symbionts

**DOI:** 10.1371/journal.pone.0114865

**Published:** 2014-12-09

**Authors:** Eugen Bauer, Hassan Salem, Manja Marz, Heiko Vogel, Martin Kaltenpoth

**Affiliations:** 1 Insect Symbiosis Research Group, Max Planck Institute for Chemical Ecology, Jena, 07745, Germany; 2 Faculty of Mathematics and Computer Science, Friedrich Schiller University, Jena, 07743, Germany; 3 Department of Entomology, Max Planck Institute for Chemical Ecology, Jena, 07745, Germany; International Atomic Energy Agency, Austria

## Abstract

The acquisition and vertical transmission of bacterial symbionts plays an important role in insect evolution and ecology. However, the molecular mechanisms underlying the stable maintenance and control of mutualistic bacteria remain poorly understood. The cotton stainer *Dysdercus fasciatus* harbours the actinobacterial symbionts *Coriobacterium glomerans* and *Gordonibacter* sp. in its midgut. The symbionts supplement limiting B vitamins and thereby significantly contribute to the host’s fitness. In this study, we experimentally disrupted the symbionts’ vertical transmission route and performed comparative transcriptomic analyses of genes expressed in the gut of aposymbiotic (symbiont-free) and control individuals to study the host immune response in presence and absence of the mutualists. Annotation of assembled cDNA reads identified a considerable number of genes involved in the innate immune system, including different protein isoforms of several immune effector proteins (specifically i-type lysozyme, defensin, hemiptericin, and pyrrhocoricin), suggesting the possibility for a highly differentiated response towards the complex resident microbial community. Gene expression analyses revealed a constitutive expression of transcripts involved in signal transduction of the main insect immune pathways, but differential expression of certain antimicrobial peptide genes. Specifically, qPCRs confirmed the significant down-regulation of c-type lysozyme and up-regulation of hemiptericin in aposymbiotic individuals. The high expression of c-type lysozyme in symbiont-containing bugs may serve to lyse symbiont cells and thereby harvest B-vitamins that are necessary for subsistence on the deficient diet of Malvales seeds. Our findings suggest a sophisticated host response to perturbation of the symbiotic gut microbiota, indicating that the innate immune system not only plays an important role in combating pathogens, but also serves as a communication interface between host and symbionts.

## Introduction

One of the reasons for the ecological success of insects is the acquisition and vertical transmission of bacterial symbionts [1]. These mutualists can aid in the digestion of complex dietary polymers [2], offer nutritional supplementation [3] or act as a defensive barrier against intruding pathogens [4]. In particular, intestinal associations with microorganisms have been shown to be beneficial for the host by the provision of nutrients important for growth [5]. Controlling and maintaining these populations of gut microbes is a challenging task for the host: While certain microorganisms are beneficial, others are detrimental and can lead to fitness disadvantages. Moreover, under certain conditions even microbes which are otherwise beneficial can cause damage [6]. Therefore, the host needs a regulatory system to keep symbiont populations in line. This fine-tuned regulation is realized by the innate immune system, which serves as a communication interface between host and symbiont to guide and modulate cooperative interactions [7].

In insects, the humoral immune response is triggered primarily by pattern-recognition proteins, which identify specific microbial surface compounds, e.g. peptidoglycans [8]. After this recognition step, several immune pathways can be induced. Signal transduction via the Toll, Imd and JAK/STAT pathways lead to the expression of immune effector proteins such as antimicrobial peptides (AMPs), which act directly against the invading microorganisms [9]. In addition, phenoloxidase activation by serine proteases can lead to an adaptive response by encapsulation of foreign cells [10].

While the molecular basis of host-pathogen interactions has received considerable attention and is reasonably well understood, particularly in *Drosophila melanogaster* [11], [12], few studies have addressed the molecular nature of the insect immune response towards beneficial gut symbionts [13]. These analyses show the differential expression of antimicrobial peptides in response to symbiont perturbation. In particular, c-type lysozyme and defensin-like genes were upregulated in the stinkbug *Riptortus pedestris*, when bacterial symbionts localized in midgut crypts were experimentally removed to create aposymbiotic individuals [13]. However, studies based on nutritionally relevant gut symbionts without a specialized localization are still lacking, partially because insect models which allow the targeted experimental removal of intestinal microorganisms from complex communities are scarce.

Within the Pyrrhocoridae (Hemiptera), characterization of the intestinal microbial community revealed a consistent and conserved microbiota, with the co-occurrence of two actinobacterial taxa belonging to the family Coriobacteriaceae (*Coriobacterium glomerans* and *Gordonibacter* sp.) [14], [15]. Similar to other hemipteran insects [16], [17], firebugs rely on an extracellular post-hatch mechanism for the vertical transmission of their actinobacterial symbionts through the deposition of bacteria-containing fecal droplets by adult females over newly laid eggs, with the subsequent probing and uptake of the symbionts by the hatched nymphs [18]. In the European firebug (*Pyrrhocoris apterus*) and the African cotton stainer (*Dysdercus fasciatus*) (both Pyrrhocoridae), sterilization of the egg surface results in aposymbiotic individuals, which lack the actinobacterial symbionts [18]. Such individuals show slower growth rates, higher mortality and lower reproductive success [15], which indicates that the actinobacterial symbionts contribute an essential function towards their insect host. A recent study demonstrated that the fitness contribution of the symbionts lies in the nutritional provisioning of B-vitamins to the host, and transcriptomic analyses revealed a tight integration of the symbionts into the host’s vitamin metabolism [19].

In this study, we investigated the immune response of *D. fasciatus* with respect to symbiont perturbation by a comparative transcriptomic analysis of midgut samples from aposymbiotic and symbiotic individuals. We accompany the expression analysis with qPCR validations and phylogenetic analyses of key genes involved in the response to symbiosis such as hemiptericin and defensin. The results of this study provide first insights into the host immune factors involved in the maintenance of this complex and specific extracellular gut microbiota.

## Materials and Methods

### Insect rearing and ethics statement

Live specimens of *Dysdercus fasciatus* were obtained from a laboratory culture at the University of Würzburg, Germany, and reared at the Max Planck Institute for Chemical Ecology, Jena, Germany. The insects were reared in plastic containers (20×35×22 cm) at a constant temperature of 28°C and exposed to long light regimes (16 h/8 h light/dark cycles). The bugs were provided *ad libitum* with previously autoclaved water and dry cotton seeds (*Gossypium barbadense*).

### Experimental elimination of the Coriobacteriaceae symbionts

The generation of aposymbiotic bugs involved the sterilization of egg surfaces following the procedure described previously by Salem *et al* [15]. Briefly, three day old eggs were submerged in ethanol for 5 minutes, followed by bleach (12% NaOCl) for 45 seconds. Subsequently, residual bleach was removed by washing several times in water. Untreated eggs served as symbiont-containing controls, as it was previously shown that the egg surface sterilization procedure itself does not adversely affect host fitness [15]. To examine the differential pattern of host gene expression in response to symbiont elimination on the natural diet of cotton seeds, five egg clutches of *D. fasciatus* were harvested, and each clutch was separated into two experimental treatments and reared on cotton seeds: (i) symbiotic (untreated), and (ii) aposymbiotic (egg surface sterilized). This design was chosen to reduce the potential influence of genetic variability among host egg clutches and allowed the use of paired statistical tests for analysis.

### RNA extraction and reverse transcription

Three days following adult emergence, a single individual was collected from every experimental treatment replicate, and through dissection, its midgut region (M1–M4) was harvested. Once dissected, the midgut was stabilized in RNA*later* solution (Qiagen) and stored at −20°C. Total RNA isolations were performed using the RNeasy Micro Kit (Qiagen) following the manufacturer’s guidelines. Integrity and quality of the RNA samples were determined using the RNA 6000 Nano LabChip kit (Agilent Technologies) on an Agilent 2100 Bioanalyzer (Agilent Technologies) according to the manufacturer’s instructions. cDNA was then generated with the QuantiTect Reverse Transcription kit (Qiagen) using the included RT Primer Mix according to the manufacturer’s guidelines. To account for possible shortcomings during RNA extraction and reverse transcription and to confirm the success of symbiont elimination after eggs surface sterilization, diagnostic PCR screens targeting the host’s 18S rRNA gene and the Coriobacteriaceae symbionts’ 16S rRNA genes were conducted using the generated cDNA according to procedures described previously [15].

### Transcriptome sequencing and library construction

Sequencing was conducted using RNA extractions from dissected whole midgut regions (M1–M4) of symbiotic and aposymbiotic bugs fed on their natural diet of cottonseeds. RNA from five individuals (one per egg clutch) of each treatment was pooled, resulting in two samples.

Prior to sequencing, the extracted RNA was exposed to rRNA depletion to minimize the amount of ribosomal RNA in the final samples (Analytik Jena, Jena, Germany). Additionally, a poly-A enrichment strategy was used to separately enrich for eukaryotic and prokaryotic RNA, respectively (Analytik Jena, Jena, Germany). Sequencing of the resulting four samples (poly-A enriched and depleted fractions of symbiotic and aposymbiotic bugs, respectively) was performed by a commercial service provider (Fasteris; http://www.fasteris.com) using 5 µg total cDNA (per sample) on the HiSeq 2000 Sequencing System from Illumina (http://www.illumina.com/webcite), utilizing the paired-end 100 bp technology with the TruSeq SBS Kit v3 on a HiSeq Flow Cell v3. The sequence data generated in this study have been deposited at the European Nucleotide Archive in the Sequence Read Archive database under accession number PRJEB6171. The complete study can be directly accessed under http://www.ebi.ac.uk/ena/data/view/PRJEB6171.

The paired-end reads were trimmed according to the sequencing quality test using Trimmomatic (v0.30) [20]. The leading and trailing bases of each read were cut off if the quality values were below the default threshold. Additionally, reads were discarded if they were shorter than 30 base pairs after trimming. Following quality checks, the trimmed reads were assembled *de novo* into contigs using Trinity (r2012-10-05) [21]. The minimal contig length was set to 200 and the *k-mer* length to 25 base pairs. The read libraries of the poly-A depleted and enriched treatments were separately pooled and assembled into backbones, respectively. After the assembly with Trinity, the contigs were clustered by CD-HIT EST (v4.5.7) [22] according to their sequence similarity to remove potential duplicates. Sequences with more than 99% sequence similarity to other contigs were subsequently collapsed. For the assignment of expression values to each constructed transcript in the respective library, the original reads were mapped back to the respective backbone assembly using Bowtie2 (v2.0.3) [23]. The generated output was processed using SAMtools (v0.1.18) [24] to create BAM files and assess the coverage depth as the number of reads mapped to each transcript using the R package Rsamtools (v1.14.1).

The expression information was normalized using the RPKM (reads per kilobase of transcript per million of mapped reads) transformation to obtain estimates of relative expression levels. Homology searches (BLASTx and BLASTn) of unique contig sequences and functional annotation by gene ontology terms (GO; http://www.geneontology.org) were conducted with an E-value cutoff of 10^−6^ using the BLAST2GO software suite v2.4.1 (http://www.blast2go.de).

Based on the taxonomic annotations of the BLAST search results from the poly-A depleted dataset, we classified different bacterial groups on the order level using MEGAN (v4) [25]. To obtain the relative abundance, we calculated for each bacterial group the overall sum of mapped reads divided by the total number of bacterial reads mapped to the specific group.

In the ploy-A enriched dataset, transcripts with annotations related to the innate immune response were extracted using specific GO terms. Additionally, sequences representing antimicrobial peptides were detected using a bi-directional local BLAST search (E-value cutoff of 10^−5^) with a compiled database consisting of publicly available antimicrobial peptide sequences in Genbank from various insects.

### Phylogenetic analyses of selected transcripts

Phylogenetic analyses were performed with the software tool MEGA5 [26] for selected transcripts with the same annotation, as obtained from the NCBI database. The coding regions of the nucleotide sequences were first determined and translated into protein sequences using Augustus (v2.7) [27]. The protein sequences were subsequently aligned with Muscle [28]. Maximum likelihood trees were then constructed with 500 bootstrap replicates.

### Validation of host gene expression by quantitative PCR

Quantitative PCRs (qPCRs) for the candidate host immune effector genes were conducted across the individual RNA samples from aposymbiotic and symbiotic bugs used for sequencing (five replicate individuals per treatment) to confirm the transcriptome sequencing results. Primers were designed based on the candidate gene sequences of hemiptericin, defensin, c- and i-type lysozyme (S1 Table) from the transcriptome and checked for specificity *in vitro* using capillary sequencing of amplified PCR products as described previously [15]. For pyrrhocoricin, no sufficiently specific and efficient qPCR assay could be designed, based on the short transcript sequence available, so this gene was not included in the qPCR analysis. Conditions for qPCR were optimized using a VWR Gradient Thermocycler (VWR, Radnor, PA, USA) at various annealing temperatures (60–68°C).

The qPCR reactions were performed using a RotorGene-Q cycler (Qiagen, Hilden, Germany). The final reaction volume of 25 µl included the following components: 1 µl of cDNA template, 2.5 µl of each primer (10 µM), 6.5 µl of autoclaved distilled H_2_O, and 12.5 µl of SYBR Green Mix (Qiagen, Hilden, Germany). Standard curves for absolute quantification in the qPCR (10-fold dilution series from 1 ng/µl to 10^−6 ^ng/µl) were generated using purified PCR products for all primer pairs after measuring the PCR product concentrations using a NanoDrop1000 spectrophotometer (Peqlab, Erlangen, Germany). For qPCR, the following cycling parameters were used: 95°C for 10 min, followed by 45 cycles of 68°C for 30 s, 72°C for 20 s, and 95°C for 15 s. Subsequently, a melting curve analysis was conducted by increasing the temperature from 60°C to 95°C within 20 min. Six replicates of one of the standard concentrations were used, for each primer pair and concentration, for the configuration and calibration of the standard curve. The resulting averages were then utilized to correct for possible errors in the DNA concentration measurements. Based on the standard curve, absolute transcript copy numbers were calculated according to [29].

### Statistical analysis

The absolute expression determined by qPCR was normalized with the expression of a housekeeping gene (60S ribosomal protein L13a). The normalized expression levels in the aposymbiotic and symbiotic treatments were then compared using the Wilcoxon signed rank test to assess levels of significance (P<0.05).

## Results

### Success of symbiont elimination

Sterilization of egg surfaces resulted in aposymbiotic firebugs that were free of both Coriobacteriaceae symbionts, as confirmed by *C. glomerans*- and *Gordonibacter*-specific diagnostic PCRs. Conversely, symbiotic bugs tested positive for both bacterial species (data not shown).

### Bacterial transcripts in the midgut of *D. fasciatus*

**Figure 1:**
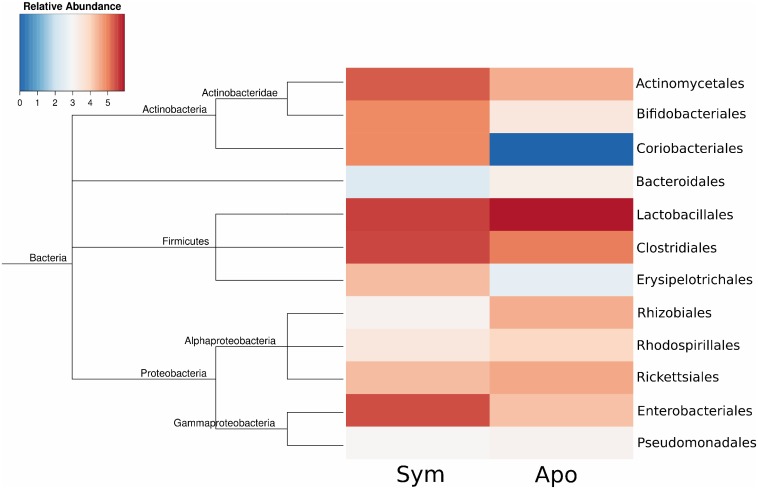
Heatmap of the sum of mapped reads (logarithmic scale) on bacterial transcripts annotated as the depicted taxonomic groups. The tree on the left represents the phylogenetic relationships of the bacteria based on the NCBI database.

In total, RNA sequencing of the poly-A depleted treatments yielded 38,207,274 reads with a combined length of 3.8 Gbp. After trimming, backbone assembly and subsequent clustering of similar sequences resulted in 89,807 contigs with a N50 length of 955 base pairs. Despite the poly-A separation strategy, only a small subset of bacterial transcripts (1,651 contigs) were recovered and annotated in the poly-A depleted fraction (Fig. 1), which, in turn, failed to provide a coherent overview of the microbiota’s metabolic and cellular processes in *D. fasciatus’* midgut. However, the transcriptional signal was still indicative of the taxonomic diversity of metabolically active bacterial species within the midgut of symbiotic and aposymbiotic firebugs.

Overall, the expression patterns between aposymbiotic and symbiotic bugs were highly similar with minor differences in Lactobacillales, Rickettsiales and Rhizobiales. The strongest difference was found in relation to transcripts belonging to the Coriobacteriaceae symbionts (*C. glomerans* and *Gordonibacter sp*.). As expected and in contrast to the high expression patterns observed in symbiotic bugs, no Coriobacteriaceae transcripts were retrieved from the aposymbiotic treatment.

### Immune system-related transcripts in the midgut transcriptome of *D. fasciatus*

In total, RNA sequencing of the poly-A enriched treatments yielded 36,456,544 reads with a combined length of 3.6 Gbp. After trimming, backbone assembly and subsequent clustering of similar sequences resulted in 55,222 contigs with a N50 length of 1,411 base pairs. Of those contigs, 20,174 received an annotation via BLAST homology. The majority of the annotations were based on hits from *Riptortus pedestris* and *Acyrthosiphon pisum*, the bean bug and pea aphid, respectively. From the BLAST results, 3,362 contigs were assigned to functional categories according to gene ontology (GO), most of which were determined to be involved in cellular and metabolic processes and biological regulation.

**Figure 2:**
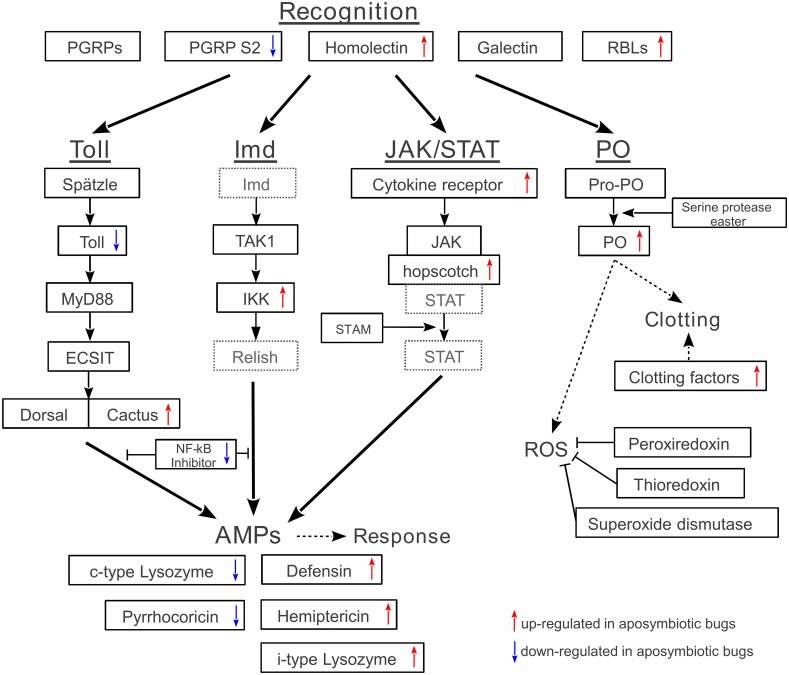
Overview of immune response related transcripts with their respective regulation pattern. Detected transcripts for the different immune pathways are indicated in solid boxes, relevant proteins in the pathway that were not found are depicted in dashed boxes. Transcripts with more than twofold changes in expression between symbiotic and aposymbiotic bugs are indicated by arrows (red  =  up-regulated in aposymbiotic bugs, blue  =  down-regulated in aposymbiotic bugs).

Here we focus on gene candidates involved in immune-system processes of the insect host (Fig. 2), recovered from the poly-A enriched treatment. Four genes putatively responsible for recognition, and 20 genes involved in signal transduction in the major insect immune pathways Toll, Imd, JAK/STAT, and phenoloxidase were recovered (Table 1, Fig. 2). With nine annotated transcripts, the Toll pathway constituted the most comprehensive set, whereas the Imd and JAK/STAT pathways contained significantly fewer annotated transcripts (both four transcripts). For the phenoloxidase pathway, three transcripts were assigned as pro-phenoloxidase (Dfas-51289), serine protease easter-like (Dfas-14165), and mature phenoloxidase (Dfas-45148).

**Table 1:** Annotation and normalized expression values (RPKM) of transcripts putatively involved in recognition and signalling of the innate immune response.

			Normalized expression(RPKM)		Foldchange
Sequence ID	Annotation	E-value	Symbiotic	Aposymbiotic	(Sym/Apo)
**Recognition**					
Dfas-12970	Cohesin subunit sa-1-like	6.8E-8	1.39	2.50	0.56
Dfas-25404	PGRP s2-like	3.0E-16	0.39	0.13	2.96*
Dfas-27487	Hemolectin cg7002-pa	1.1E-10	0.00	0.33	N/A
Dfas-45194	Galectin partial	2.5E-8	1.47	2.12	0.69
**Toll Pathway**					
Dfas-02931	Ecsit_drome ame	1.0E-101	4.06	3.76	1.08
Dfas-49270	Myd88 protein	7.3E-44	47.44	48.31	0.98
Dfas-17230	Serpin 38f	4.6E-10	7.22	15.29	0.47*
Dfas-03438	Partial spaetzle precursor	8.2E-27	10.27	12.48	0.82
Dfas-17931	Protein spaetzle	4.5E-19	2.59	4.52	0.57
Dfas-01522	Protein toll	7.3E-31	0.70	0.24	2.96*
Dfas-16185	Cactus	6.0E-44	10.89	42.43	0.26*
Dfas-36948	Dorsal 1c	1.8E-6	0.78	0.79	0.99
Dfas-25024	Mapkk kinase dsor1	0	2.33	4.84	0.48*
**IMD pathway**					
Dfas-48512	Nf kappa b inhibitor	8.8E-12	1.60	0.36	4.44*
Dfas-03678	Tp53-regulated inhibitor	7.2E-21	4.05	5.62	0.72
Dfas-30553	Tak1-associated binding protein	1.9E-12	1.45	1.42	1.02
Dfas-48614	Elongator complex protein 1	1.1E-85	0.86	2.26	0.38*
**JAK/STAT pathway**					
Dfas-28681	Tyrosine-protein kinase jak2	4.3E-19	0.00	0.36	N/A
Dfas-39587	Tyrosine-protein kinasehopscotch	1.4E-38	0.86	0.44	1.97
Dfas-35575	Cytokine receptor	0	1.02	3.15	0.32*
Dfas-43095	Signal transducing adaptermolecule	2.8E-135	15.34	20.14	0.76
**Phenoloxidase pathway**					
Dfas-51289	Pro-phenol oxidase subunit 2	1.8E-28	0.58	0.79	0.74
Dfas-45148	Phenoloxidase subunit a3-like	1.3E-44	0.42	0.84	0.49*
Dfas-14165	Serine protease easter-like	5.7E-41	6.85	7.68	0.89
Dfas-26420	Melanization-related protein	1.6E-29	0.00	1.21	N/A
Dfas-50961	Limulus clotting factor c	3.9E-35	0.19	2.07	0.09*

Differentially expressed genes (>2 fold) are marked with asterisks.

The expression of transcripts presumably involved in recognition and signaling generally showed relatively low expression levels based on the normalized RPKM values (Table 1). The low expression pattern of these transcripts nonetheless pointed towards an up-regulation (Fold >2) in aposymbiotic individuals (Table 1), with the exception of the transcripts annotated as protein toll (Dfas-01522), nf-kappa b inhibitor (Dfas-48512) and peptidoglycan recognition protein s2 (Dfas-25404), which were up-regulated (Fold >2) in symbiotic bugs. Most of the transcripts potentially involved in signaling showed no differential expression at all.

### Antimicrobial peptide transcripts

Among the immune effector genes, several AMP types with potential isoforms were detected (Table 2): 11 transcripts were annotated as hemiptericin (Dfas-21661, Dfas-00011, Dfas-50935, Dfas-16990, Dfas-33450, Dfas-43693, Dfas-01725, Dfas-21700, Dfas-46208, Dfas-03942, Dfas-25640), three as defensin-like genes (Dfas-02709, Dfas-51099, Dfas-33854), two as pyrrhocoricin (Dfas-00911, Dfas-33105), two as i-type lysozyme (Dfas-45802, Dfas-30083), and one as c-type lysozyme (Dfas-30397). The immune effector genes generally showed much higher expression levels than genes putatively involved in immune signaling and microbe recognition (Table 1 and 2). Comparison of AMP expression between symbiotic and aposybiotic bugs revealed that most of the detected transcripts were differentially expressed (Fold >2), with some genes being up- and others down-regulated in response to symbiont elimination (Table 2). Among the transcripts up-regulated in aposymbionts were i-type lysozyme, hemiptericin, and defensin, whereas pyrrhocoricin and c-type lysozyme were down-regulated in symbiont-deprived bugs. Within each AMP, the expression of the isoforms was relatively consistent, with only minor variations in the RPKM values.

**Table 2:** Annotation and normalized expression values (RPKM) of putative antimicrobial peptide transcripts.

			Normalized expression (RPKM)		Fold change
Sequence ID	Annotation	E-value	Symbiotic	Aposymbiotic	(Sym/Apo)
Dfas-45802	Lysozyme i-type	2.6E-43	1.27	2.57	0.49*
Dfas-30083	Lysozyme i-type	5.2E-46	0.84	2.38	0.35*
Dfas-30397	Lysozyme c-type	9.5E-41	649.66	130.97	4.96*
Dfas-21661	Hemiptericin	2.9E-57	1.86	2.52	0.74
Dfas-00011	Hemiptericin	1.9E-80	0.82	7.02	0.12*
Dfas-50935	Hemiptericin	5.9E-53	0.89	1.81	0.49*
Dfas-16990	Hemiptericin	1.8E-53	0.39	5.09	0.08*
Dfas-33450	Hemiptericin	1.0E-80	1.20	7.00	0.17*
Dfas-43693	Hemiptericin	1.4E-36	0.00	0.28	N/A
Dfas-01725	Hemiptericin	1.1E-86	1.60	8.25	0.19*
Dfas-21700	Hemiptericin	1.0E-53	1.24	12.18	0.10*
Dfas-46208	Hemiptericin	2.0E-83	0.68	6.23	0.11*
Dfas-03942	Hemiptericin	7.5E-87	1.00	3.37	0.30*
Dfas-25640	Hemiptericin	3.0E-54	3.08	5.28	0.58
Dfas-02709	Defensin 4	2.0E-7	2.40	1.62	1.48
Dfas-51099	Defensin a	2.6E-25	0.86	1.45	0.59
Dfas-33854	Defensin-2 precursor	3.6e-20	1.10	3.23	0.34*
Dfas-00911	Pyrrhocoricin	2.2e-10	36.77	19.81	1.86
Dfas-33105	Pyrrhocoricin	2.0e-10	19.91	8.62	2.31*

Differentially expressed genes (>2 fold) are marked with asterisks.

### Expression validation of antimicrobial peptides with qPCR

**Figure 3:**
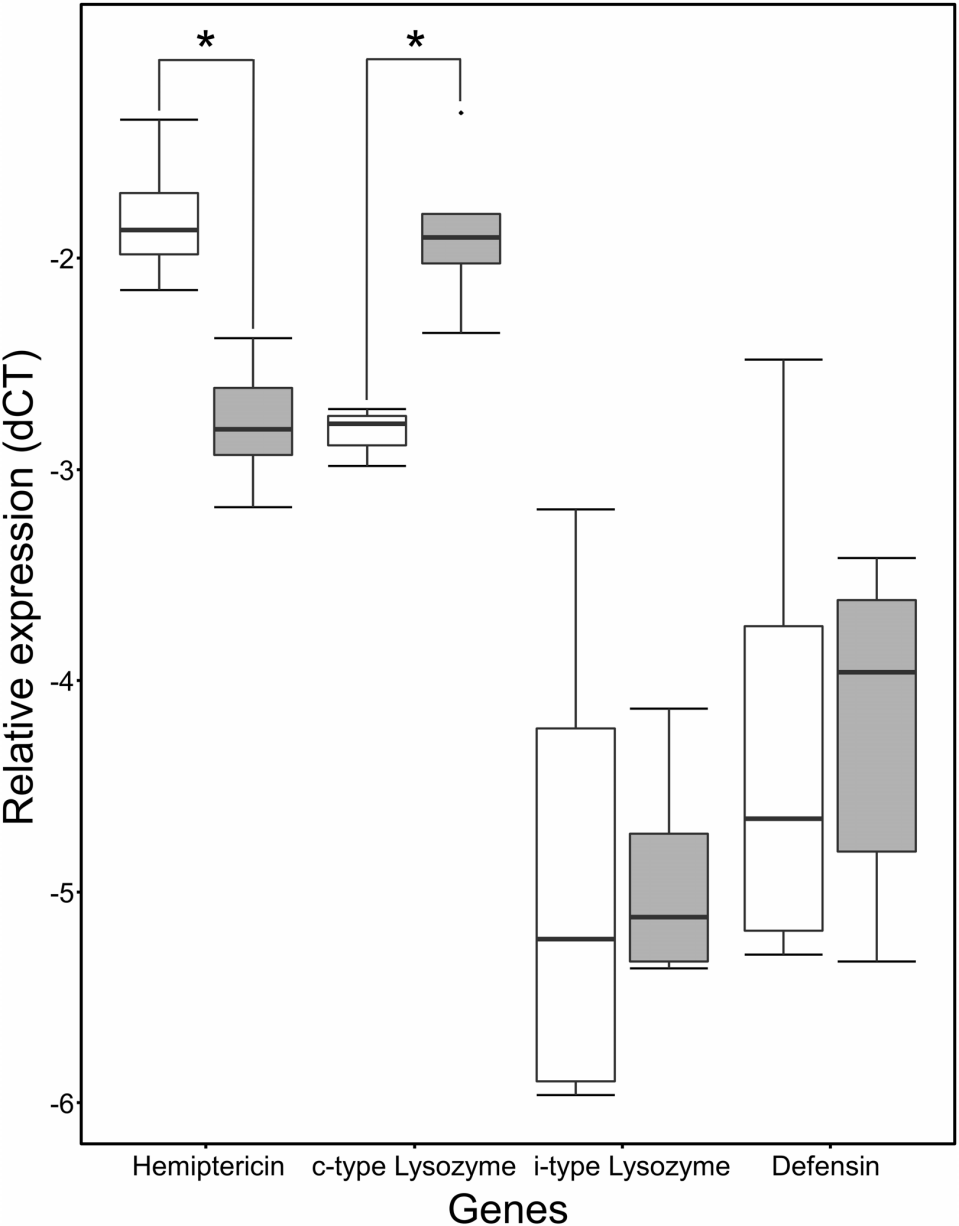
Relative expression of antimicrobial peptides in symbiotic and aposymbiotic bugs based on qPCR experiments. Expression values were calculated as the dCT based on the expression of a housekeeping gene (60S ribosomal protein L13a). Grey boxes represent expression values of symbiotic bugs, open boxes expression values of aposymbionts. Significant differential expression is indicated with an asterisk (Wilcoxon signed rank tests, p<0.05).

To validate the observed normalized expression patterns of c- and i-type lysozyme, defensin and hemiptericin (Table 2), quantitative PCRs were performed (Fig. 3). The same expression patterns were observed for hemiptericin and c-type lysozyme compared to the RNAseq expression analysis. The expression of hemiptericin showed a significant up-regulation in aposymbiotic individuals (Wilcoxon signed rank test, p<0.01), whereas the gene for c-type lysozyme was significantly down-regulated in aposymbiotic bugs (Wilcoxon signed rank test, p<0.05). For defensin and i-type lysozyme, no significant differences could be detected in the qPCRs.

### Phylogenetic analysis of transcripts encoding antimicrobial peptides

**Figure 4:**
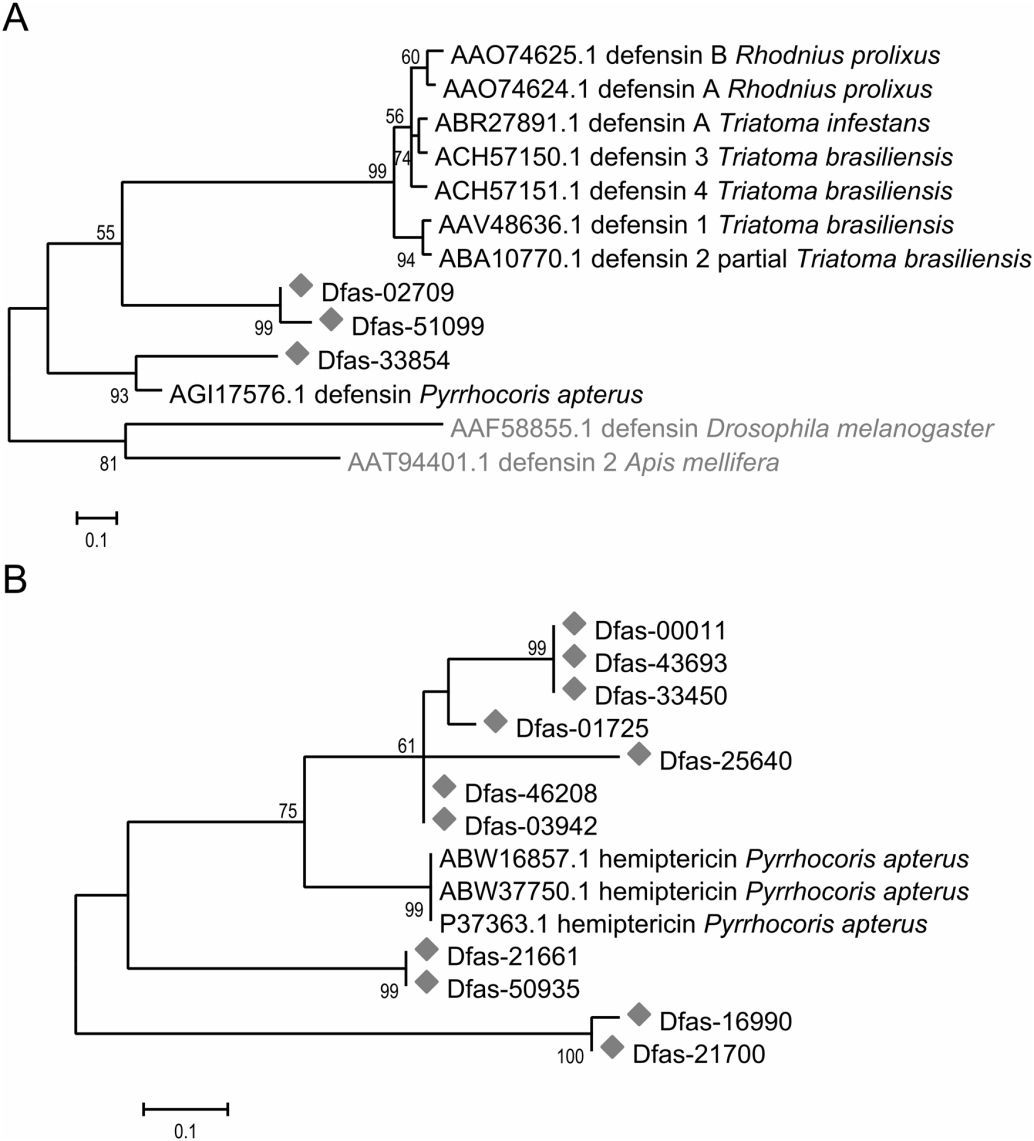
Maximum likelihood phylogenetic trees of defensin (A) and hemiptericin (B) transcripts. Sequences recovered in this study are marked with a grey diamond, sequences used as an outgroup are marked in grey font. Bootstrap values above 50% are given at the nodes (based on 500 replicates).

To study the micro-diversity of sequences annotated as defensin and hemiptericin, phylogenetic analyses were performed (Fig. 4). For both genes, sequence alignments revealed various amino acid substitutions across the different isoforms (S1 and S2 Figures). Defensin-related transcripts showed two distinct isoforms, one of which clustered with a published sequence for *P. apterus* (Fig. 4A). For hemiptericin, phylogenetic analysis revealed three distinct isoforms (Fig. 4B), one of which clustered together with sequences of *P. apterus* obtained from the GenBank database. The transcript annotated as c-type lysozyme (Table 2) was highly similar to the *R. pedestris* lysozyme sequence (S3 Figure).

## Discussion

In this study, we conducted a comparative transcriptomic analysis of midgut samples from symbiont-containing and aposymbiotic individuals of the cotton stainer, *D. fasciatus*. We identified a number of differentially expressed transcripts involved in the immune response of the host following symbiont elimination (Fig. 2). These results provide first insights into the molecular interactions between cotton stainers and their extracellular intestinal symbionts and complement the knowledge of immune responses in perturbed gut ecosystems of insects [30]. In the following paragraphs, we will discuss the putative biological roles of the immune related genes identified in this study and the implications of their differential expression upon manipulation of the microbial midgut community in *D. fasciatus.*

### Effect of symbiont elimination on the gut microbiota

The few collected transcripts of bacterial taxa (Fig. 1) were insufficient to provide a comprehensive view of the microbial gene expression. However, they did provide significant insights into the taxonomic composition of metabolically active bacterial symbionts in the midgut of *D. fasciatus*. Our analysis revealed a microbial community that is largely consistent with earlier results characterizing the gut microbiota of firebugs, with high abundances of Actinobacteria, Firmicutes, and Proteobacteria [14], [15]. Most importantly, bacteria of the family Coriobacteriaceae were abundant in symbiotic bugs, but completely absent from aposymbiotic individuals. These results augment previous findings [15] demonstrating that the egg surface sterilization is specific towards ridding firebugs from their association with *C. glomerans* and *Gordonibacter* sp.

Concerning the distribution of other microbial taxa, only minor quantitative differences between aposymbiotic and symbiotic bugs were detected (Fig. 1). This indicates that even though a major group of mutualistic bacteria were removed from the intestinal ecosystem, the host was still able to maintain a specific composition of the remaining microbiota, by providing a selective environment and/or by regulation via the immune system. The minor differences in gene expression of transcripts belonging to Lactobacillales, Rickettsiales and Rhizobiales might be in this respect indicative for a compensatory mechanism from the host side.

### Constitutive expression of immune signaling pathways

We identified a subset of the known pattern recognition proteins (Table 1) involved in the sensing of foreign organisms [8]. Among those was one short type peptidoglycan recognition protein (PGRP s2-like). In *Drosophila*, short class peptidoglycan recognition proteins are responsible for the detection of Gram-positive bacteria and subsequent induction of immune pathways [31]. Thus, this protein may be involved in the recognition of the mutualistic Coriobacteriaceae in *D. fasciatus*, which is consistent with the up-regulation in symbiotic as compared to aposymbiotic bugs (Table 1).

Among the immune-related transcripts detected in this study (Table 1, Fig. 2), all four major insect immune pathways [8] were recovered in *D. fasciatus*. The detected Toll pathway provided the most comprehensive view, with annotated transcripts spanning the signal transduction cascade from precursor protein *spaetzle* to the transcription factors *dorsal* and *cactus* [32]. In contrast, the detected genes involved in the Imd pathway provide only a fragmented view of this signaling cascade. The scarcity of annotated Imd-pathway genes is likely due to the absence of many Imd genes in the pea aphid *Acyrthosiphon pisum* [33], which is the closest organism to the Pyrrhocoridae with a sequenced genome available, and thus, with reliable homology information. Across the four major pathways (Toll, Imd, JAK/STAT, phenoloxidase), gene expression was generally low. Furthermore, no distinct regulation patterns could be detected, with only few genes being differentially expressed (>2 fold) between symbiotic and aposymbiotic individuals. These observations are in line with other studies, demonstrating the constitutive expression of immune signaling pathway genes [34].

### Expression patterns of immune effectors depict differentiated biological roles

In contrast to the low and constitutive expression of pathway-internal genes, the transcripts of the end products of signal transduction, the immune effector genes, show generally higher expression levels that differ between symbiotic and aposymbiotic bugs (Table 2). Considering that antimicrobial peptides (AMPs) are the front line of defense against microbial pathogens [9], this indicates a highly specific as well as regulated immune response towards symbiont perturbation.

The expression patterns across the detected antimicrobial peptides differed considerably (Table 2). In general, two groups could be differentiated: i) Transcripts down-regulated in aposymbiotic bugs comprised c-type lysozyme and pyrrhocoricin, whereas ii) defensin, hemiptericin and i-type lysozyme were up-regulated upon symbiont elimination. Validation of expression patterns via qPCR (Fig. 3) confirmed the differential expression for c-type lysozyme and hemiptericin, but not for defensin and i-type lysozyme across the studied individuals. Therefore, c-type lysozyme and hemiptericin were considered strong candidates for an important role in responding towards perturbation of the microbial midgut community. Additionally, pyrrhocoricin represents an interesting candidate gene with potential down-regulation in aposymbiotic bugs, but the lack of a specific qPCR assay prevented us from gaining more detailed insights on the consistency of the expression patterns across replicate individuals.

The high expression of c-type lysozyme (Fig. 3) and pyrrhocoricin (Table 2) in symbiotic bugs might serve to regulate the association with the pivotal Coriobacteriaceae symbionts. This idea is supported by the primary activity of both lysozymes [35] and pyrrhocoricin [36] against Gram-positive bacteria. In weevils of the genus *Sitophilus*, the expression of a single antimicrobial peptide gene (coleoptericin A [*colA*]) was found to be important in controlling population dynamics of mutualistic bacteria, which overreplicated in the host, when *colA* was knocked down with RNA interference [6]. The same might be true in Pyrrhocoridae species, which may require c-type lysozyme to control the abundance of the intestinal symbionts in order to maintain the host-symbiont equilibrium. This equilibrium may be particularly important for gut ecosystems, since at a higher microbial growth rate, the host might compete with its symbionts for important nutrients rather than engage in a mutualistic relationship.

Furthermore, considering the nutritional relevance of the symbiotic Coriobacteriaceae [19] up-regulation of c-type lysozyme might cause the release of nutrients via lysis of the bacterial cells [19]. In *Drosophila melanogaster,* lysozyme expression plays an important role in the digestion of intestinal bacteria [37], indicating a similar pattern as observed in this study (Fig. 3). This harvesting of nutrients through lysis of symbiont cells was also proposed in *R. pedestris* as a mechanism to release nutrients and control symbiont populations [38].

In contrast to c-type lysozyme and pyrrhocoricin, hemiptericin was significantly up-regulated in aposymbiotic individuals, and defensins as well as i-type lysozyme showed tendencies towards higher expression in aposymbiotic bugs. As in other insects, hemiptericin is known to be important in *P. apterus* for the response to foreign microorganisms, particularly Gram-negative bacteria [36]. The up-regulation upon symbiont elimination in *D. fasciatus* might therefore constitute a compensatory mechanism to control the remaining gut microbiota, which might otherwise overreplicate in the ecological niche freed by the absence of the Coriobacteriaceae.

### Isoforms of antimicrobial peptides indicate a global response to the gut microbiota

Interestingly, several isoforms of hemiptericin and defensin were detected (Fig. 4), which may allow for a fine-tuned and dynamic interaction with the intestinal community, since the different isoforms have divergent sequences (S1 and S2 Figures) and may thus act against different microbial taxa. This is particularly relevant in cotton stainers, as in addition to the Coriobacteriaceae symbionts, they consistently harbor several bacterial taxa in the Firmicutes and Proteobacteria (Fig. 1). These taxa could be differentially regulated in their abundance by individual AMPs, with the defensins primarily targeting Gram-positive and hemiptericins Gram-negative bacteria [36]. The presence of AMP isoforms has also been described for other insects [13], [39], but their role and adaptive significance in immunity remains unknown.

### Conclusions and perspectives

Using a comparative transcriptomics approach, our study revealed a differentiated immune response to the elimination of a specific group of extracellular gut symbionts. This response includes both up- and down-regulation of certain antimicrobial peptides of the host, respectively, which may serve to maintain a stable equilibrium of the complex microbiota in the host’s gut and could additionally be involved in harvesting nutrients through lysis of symbiont cells. While the transcriptomic approach provides a global view into gene expression patterns in the cotton stainer’s gut, targeted knock-down of individual immune effector genes by RNA interference [40] is necessary to provide functional insights into the interactions between the microbial symbionts and the host immune system.

## Supporting Information

S1 Figure Multiple sequence alignment of translated defensin transcripts from *D. fasciatus* (bold) and other Heteroptera.
(TIF)

S2 Figure Multiple sequence alignment of translated hemiptericin transcripts from *D. fasciatus* (bold) and *P. apterus*.
(TIF)

S3 Figure Multiple sequence alignment of translated c-type lysozyme transcripts from *D. fasciatus* (bold) and other representative insect taxa.
(TIF)

S1 Table Primers used for quantitative PCR.
(DOCX)
