# Supplementary figures and images for: Transcriptomic Immune Response of the Cotton Stainer Dysdercus fasciatus to Experimental Elimination of Vitamin-Supplementing Intestinal Symbionts
Source: PLoS One. 2014 Dec 9;9(12):e114865. doi: 10.1371/journal.pone.0114865 (PMC4260922; doi:10.1371/journal.pone.0114865)

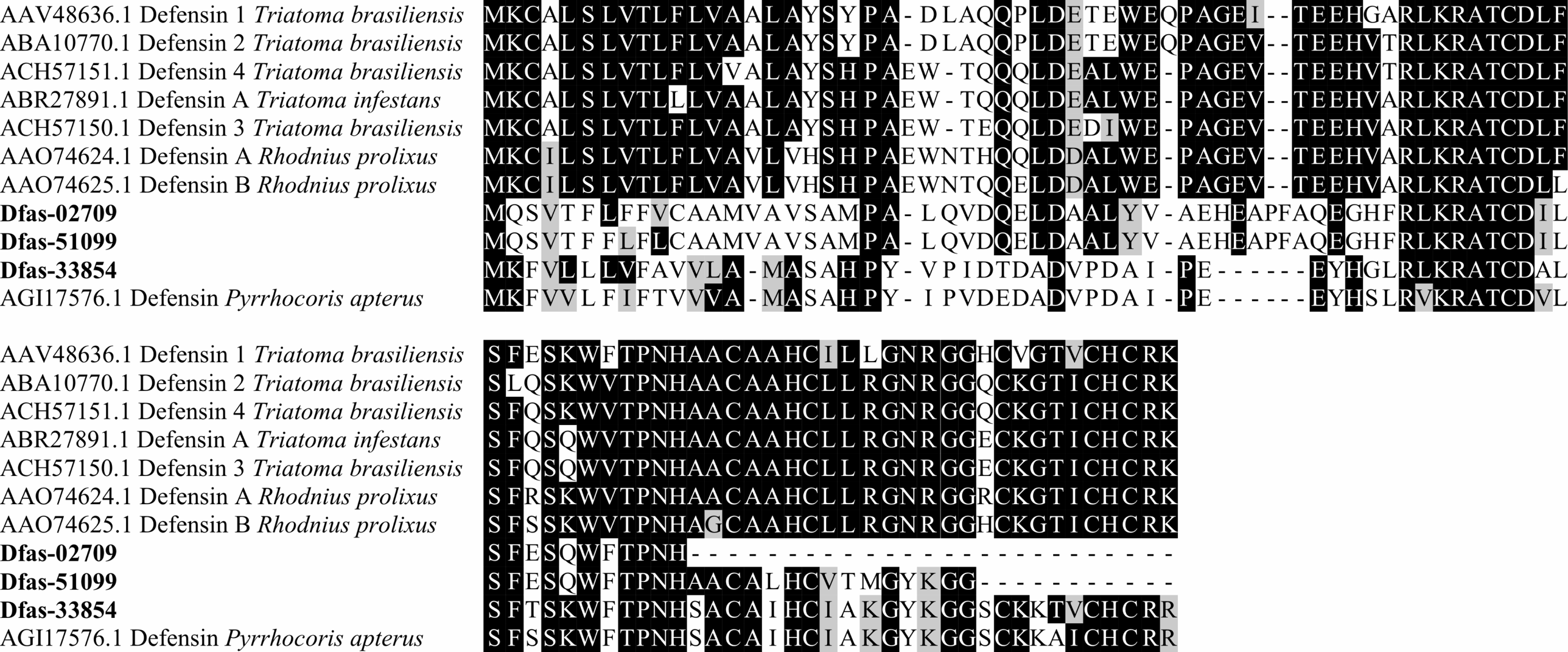

Supplement: S1 Figure — Multiple sequence alignment of translated defensin transcripts from D. fasciatus (bold) and other Heteroptera. (TIF) [file pone.0114865.s001.tif]

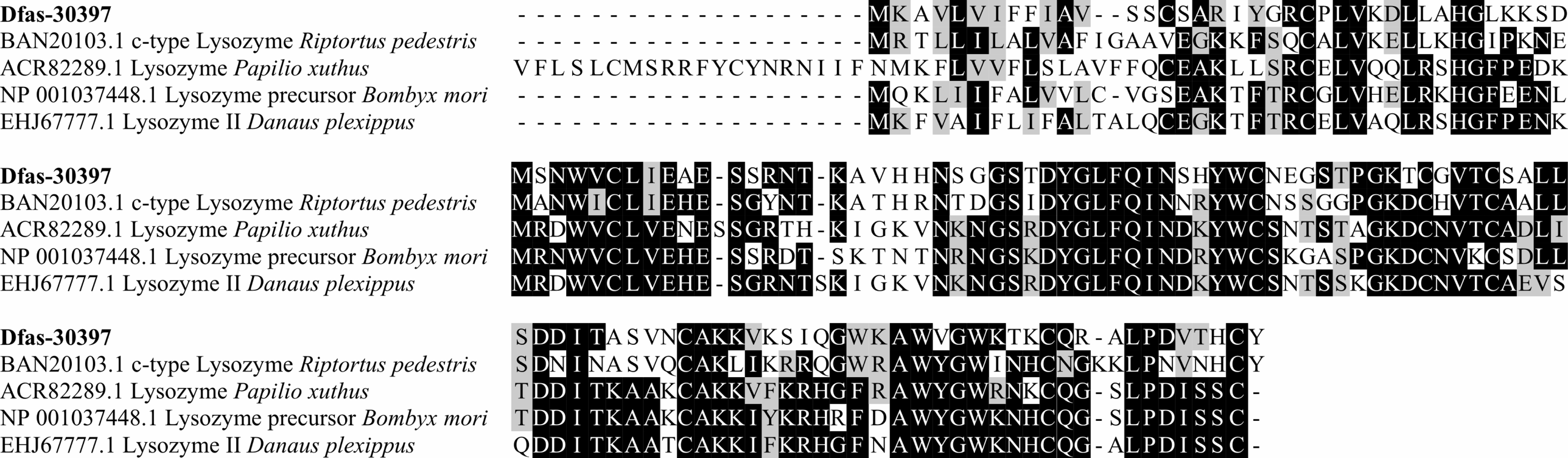

Supplement: S3 Figure — Multiple sequence alignment of translated c-type lysozyme transcripts from D. fasciatus (bold) and other representative insect taxa. (TIF) [file pone.0114865.s003.tif]
