# Supplementary material for: Transcriptomic Immune Response of the Cotton Stainer Dysdercus fasciatus to Experimental Elimination of Vitamin-Supplementing Intestinal Symbionts
Source: PLoS One. 2014 Dec 9;9(12):e114865. doi: 10.1371/journal.pone.0114865 (PMC4260922; doi:10.1371/journal.pone.0114865)
Supplement: S1 Table — Primers used for quantitative PCR. (DOCX) [file pone.0114865.s004.docx]

| **Table S1**: Primers used for quantitative PCR. | | | |
| --- | --- | --- | --- |
| **Sequence ID** | **Target** | **Primer sequence (5’ - 3’)** | **Orientation** |
| Dfas-51099 | Defensin a | GTTCTCGCACCTTCCTCCTT | fwd |
| Dfas-51099 | Defensin a | TTGTACGTAGCCGAACACGA | rev |
| Dfas-16990 | Hemiptericin | CACGAGGCTGAACGGTACTT | fwd |
| Dfas-16990 | Hemiptericin | CACCTGAGTCCTTCCGTTGT | rev |
| Dfas-45802 | Lysozyme i-type | TGTTGCAGTCCTGAGCAAAC | fwd |
| Dfas-45802 | Lysozyme i-type | CTGTCAGGGTGACGTCTGTG | rev |
| Dfas-30397 | Lysozyme c-type | TGTAGTGCCAGGATCTACGG | fwd |
| Dfas-30397 | Lysozyme c-type | TTAGCACAGTTCACCGAAGC | rev |
